# Urinary incontinence following successful closure of obstetric vesicovaginal fistula repair in Southern Ethiopia

**DOI:** 10.1186/s12905-024-02979-3

**Published:** 2024-03-07

**Authors:** Shimelis Tadesse, Desta Mekete, Shambel Negese, Dereje Zeleke Belachew, Galana Takele Namara

**Affiliations:** 1 Department of Midwifery, College of Health Science, Mattu University, P. O. Box 318, Mattu, Ethiopia; 2 Department of Midwifery, College of Medicine and Health Science, Mizan Tepi University, Mizan Tefere, Ethiopia; 3 Department of Midwifery, College of Medicine and Health Science, Ambo University, Ambo, Ethiopia

**Keywords:** Urinary incontinence, Obstetric vesicovaginal fistula, Repair outcomes, Associated factors, Southern Ethiopia

## Abstract

**Background:**

Urinary incontinence (UI) after successful closure of obstetric vesicovaginal fistula (VVF) repair is a widely recognized public health problem. However, there is insufficient research evaluating the factors associated with UI after successful obstetric VVF repair in Ethiopia.

**Objectives:**

The main aim of this study was to assess the magnitude and associated factors of UI following the successful closure of obstetric VVF repair at the Yirgalem Hamlin Fistula Center in the Sidama region of southern Ethiopia.

**Methods:**

A retrospective cross-sectional analytical study was performed on patients who underwent obstetric VVF repair at the Yirgalem Hamlin Fistula Center between 2016 and 2020. The data were collected from September to October 2021. EPI Data Version 3.1 and SPSS Version 25.0 were used for data entry and analysis. A multivariable binary logistic regression model was used for all variables significant in the bivariate binary logistic analysis to determine the association between the independent variables and outcome variables. The data are presented in tables and figures. Variables with a p-value < 0.05 were considered to be statistically associated with the study outcome.

**Results:**

In total, 499 study subjects were included. The magnitude of UI after successful closure of obstetric VVF repair was 23.25%. A Goh type 4 fistula (AOR = 4.289; 95% CI 1.431, 12.852), a fistula size > 3 cm (AOR = 8.855; 95% CI 4.786, 16.382), a partially damaged urethra (AOR = 2.810; 95% CI 1.441, 5.479), and a completely destroyed urethra (AOR = 5.829; 95% CI 2.094, 16.228) were found to be significantly associated factors with the outcome variable.

**Conclusions:**

Nearly one in four patients who had successful closure of obstetric VVF repair at the Yirgalem Hamlin fistula center had UI, which is above the WHO recommendations. The presence of a Goh type 4 fistula, large fistula size, and damaged urethral status significantly affect the presence of UI. Therefore, interventions are necessary to prevent and manage UI among patients who underwent obstetric VVF repair and had a closed fistula.

## Background

An estimated 300 million women worldwide are affected by urinary incontinence (UI). In Sub-Saharan Africa (SSA), UI is the main complication for women after obstetric vesicovaginal fistula (VVF) [1, 2]. VVF is an abnormal formation of a hole between the urinary system bladder and the reproductive system vagina [3, 4]. It is a common and devastating problem in developing countries [5]. Obstetric VVFs affect approximately 3 million women worldwide. Of these, about 2 million are found in the SSA and southern Asia. The data also illustrated that in Africa alone, 30,000–130,000 new cases develop annually [6, 7]. Likewise, an estimated 9000 patients develop obstetric fistulas each year in Ethiopia alone [8]. The lifetime prevalence rate of vesicovaginal fistula (VVF) in SSA was 3 per 1000 women of reproductive age [9]. Three possible outcomes could occur in women who underwent VVF repair. These outcomes are cured (successful closure and restored continence), successful closure but the presence of UI, and failure to close the fistula [10]. Although the successful obstetric VVF closure rate is greater than 90%, after successful obstetric VVF repair, patients complain of up to 55% UI [7]. UI after VVF closure was defined as any involuntary leakage of urine from the vagina that a patient complained about after obstetric VVF repair [11]. UI after successful obstetric VVF repair can include stress UI, overactive bladder, mixed UI, or bladder voiding disorder [12].

Despite the highest successful closure of VVF repair, UI is a common and challenging public health problem among patients who underwent VVF repair [11, 13, 14]. Patients with UI experience wetness, odor, and discomfort [15], which have significant consequences for social and emotional problems such as relationship problems, depression, and suicidal ideation [16] and can affect women’s quality of life [17–19]. This also led to patients having long waits at the center (2–4 weeks) and a burden for fistula treatment facilities or centers [20].

The prevalence of UI following successful closure of obstetric VVF repair differs among countries. For instance, in South and Middle East Asia, cross-sectional studies were conducted in India between 2011 and 2019 (18.5%) [21], Bangladesh between 2008 and 2010 (37.7%) [22], and Pakistan between 2008 and 2018 (24.5%) [12]. A retrospective cohort study in Indonesia was conducted in 2020, for which 40% of patients were affected [23].

In SSA, retrospective cohort studies demonstrated that in the Democratic Republic of Congo (DRC), between 2017 and 2019 (4.3%) [13], in Guinea (13.36%) [11], in Malawi between September 2011 and December 2014 (24%) [24]. A retrospective cross-sectional review conducted in Burkina Faso between 2010 and 2016 revealed 11.7% of patients with UI [25] and in Niger between 2011, 2013 and 2014 (63.9%) [26].

In Ethiopia, a previous cross-sectional study performed at the Bahir Dar Hamlin Fistula Center between 2013 and 2017 demonstrated that 24.8% of patients had urinary incontinence after successful closure of obstetric VVF repair [27]. Another cross-sectional study conducted at Jimma Specialized Hospital between 2011 and 2014 showed that 8.9% of UI patients were affected [8]. A previous study performed between 2008 and 2009 at Addis Ababa Fistula Hospital showed that approximately 49% of patients had UI after VVF repair [28].

Like the prevalence of UI, the risk factors also differed among the various study sites or countries. These factors included sociodemographic, obstetric, fistula, and perioperative characteristics. For example, a study performed in Guinea demonstrated that the age of the patient, duration of the fistula, presence of Goh type fistulas, shortness of the urethra, and total destruction of the bladder neck were risk factors for UI after obstetric VVF closure [11]. Another study conducted in Guinea also found that partially or totally damaged urethra status was a contributing factor for post-fistula repair UI [29]. A study performed in the DRC showed that previous history of surgery and vaginal route repair were risk factors [13]. Moreover, a study performed in India revealed that the size and site of the fistula were risk factors [21]. In addition, a study conducted in Ethiopia at the Mekelle Hamlin Fistula Center showed that patients who were younger and had their first vaginal delivery were at greater risk for UI after successful closure of VVF repair [17].

The management of UI can include lifestyle changes, physical therapy, behavioral therapy, pelvic floor exercise, medication, a sling procedure, the use of an autologous sling, or follow-up [16, 30]. During surgical repair of VVF patients, surgeons might use facial slings such as rectus slings or pubococcygeus slings, and skin grafts, which are highly important for reducing the burden of UI after successful obstetric VVF closure [20]. Overall, VVF management is individualized and depends on the surgeons skill and the application of the principles of adequate exposure, tension freeness to approximate edges, non-overlapping ping suture lines, good hemostasis, watertight closure, and enough post-repair bladder catheterization [3].

Addressing why women affected by UI after successful VVF closure has the most important clinical outcome [10] because closing the anatomical defect of VVF is not sufficient [31]. Moreover, predicting or identifying the associated factors and evaluating patients with obstetric VVF before managing them are important to avoid or prevent UI after successful obstetric VVF surgical repair [11, 12]. Despite the high prevalence of UI following successful closure of obstetric VVF repair, there is insufficient research that has assessed the factors associated with UI after successful obstetric VVF closure. Therefore, this study aimed to evaluate the prevalence and contributing factors for UI following the successful closure of obstetric VVF repair at the Yirgalem Hamlin Fistula Center in southern Ethiopia. These findings will add value to preventing UI following the successful closure of obstetric VVF repair by providing information to healthcare providers, policymakers, researchers, and governmental and nongovernmental stakeholders.

## Methods

### Study design, period, and setting

This retrospective cross-sectional observational study was conducted from September 2021 to October 2021 at the Yirgalem Hamlin Fistula Center, Southwest Ethiopia. The Yirgalem Hamlin Fistula Center is in the Sidama Regional State, 300 km away from Addis Ababa, Ethiopia. The center provides for more than 400 surgeries each for genitourinary fistulas and pelvic organ prolapse. Moreover, the center has one gynecologist, one midwife, four health officers, 14 trained aid nurses, and 38 beds [32].

### Source and study population

The source population included patients who underwent VVF surgical repair at the Yirgalem Hamlin Fistula Center, while the study populations were all patients who underwent VVF surgical repair between 2016 and 2020.

### Sample size, techniques, and procedures

All patients who underwent obstetric VVF repair between 2016 and 2020 at the Yirgalem Hamlin fistula center and met the inclusion criteria were included in this study. All medical records and the operation logbooks were reviewed for women who underwent VVF repair between January 1, 2016, and December 31, 2020. In this study, 610 study subjects were reviewed, and only 499 study subjects were included in the analysis. Other fistulas were excluded from the study due to incomplete cards, nonobstetric causes of fistulas, or other types of fistulas such as rectovaginal fistulas and concomitant vesicovaginal fistulas.

### Inclusion and exclusion criteria

This study included all vesicovaginal fistula patients who underwent repair, and the data were recorded in patients’ medical charts and operation logbooks at the Yirgalem Hamlin Fistula Center between 2016 and 2020. This study excluded all patients with concomitant rectovaginal fistula repairs, incomplete medical records, or fistulas caused by nonobstetric causes, such as congenital causes, malignant causes, or rape.

### Data collection tools and procedures

The data collection checklist was developed after reviewing previous related literature and the management format for national genitourinary fistula patients. The data were collected retrospectively from the medical charts of patients who underwent obstetric vesicovaginal fistula repair in the last five years, from January 1, 2016, to December 31, 2020. Patient sociodemographics, obstetric profiles, fistula characteristics, perioperative condition, and UI were collected.

### Study variables

The presence of urinary incontinence among patients who had successful obstetric vesicovaginal fistula repair was the dependent variable, whereas the patients’ sociodemographic, obstetric, fistula, and perioperative characteristics were the independent variables.

### Data quality control

The quality of the data was assured through training the data collectors regarding the data collection process, using a pretested data checklist, monitoring daily activities, and maintaining confidentiality.

### Data analysis

The data were entered into EPI Data Version 3.1, and the analysis was performed with SPSS version 25.0. In the univariate analysis, the data are presented as frequencies and percentages. Continuous variables are presented as the mean, range, and standard deviation (SD), and categorical variables are presented as proportions. At the bivariate level, associations between dependent and independent variables were determined using binary logistic regression. All the variables with a p-value < 0.20 at the bivariate level were included in the binary logistic regression model. Then, the crude odds ratio (COR), adjusted odds ratio (AOR), and 95% confidence interval (CI) were computed for all the variables. The level of significance was set at *p* < 0.05.

### Operational definition

Successful closure of obstetric vesicovaginal fistula repair: Women had a negative dye test or closed defect at the time of postoperative bladder catheter removal or after 21 days of repair.

Urinary incontinence after successful closure of obstetric vesicovaginal fistula repair: Women had a negative dye test but had wet urine at the time of postoperative bladder catheter removal or after 21 days of repair.

Goh-type fistulas are defined as fistulas that open from the external urinary meatus. Type 1 fistula > 3.5 cm, type 2 fistula 2.5–3.5 cm, type 3 fistula 1.5–2.5 cm, and type 4 fistula < 1.5 cm from the external urinary meatus.

The number of fistulas refers to the number of holes that develop after an abnormal connection between the bladder and the vagina, which might be one, two, or more.

## Results

### Sociodemographic characteristics of patients

Among the 499 retrospectively reviewed patients who underwent VVF repair, the majority, 245 (49.1%) were between the ages of 20 and 34 years, with a range of 14–61 years and mean age at repair of 30.74 years (SD ± 10.054). Almost two-thirds of the women, 335 (67.1%) were teenagers at their first pregnancy, with a range of 12–28 years and a mean age for first pregnancy of 17.89 years (SD ± 2.526). Similarly, the majority 301 (61.7%) of women had no formal education, and 403 (80%) were married (Table 1).

**Table 1:** Sociodemographic characteristics of patients who underwent successful closure of obstetric vesicovaginal fistula repair at the Yirgalem Hamlin fistula center, 2021 (*n* = 499)

Variables	Category	Frequency	Percentages (%)
Age at fistula repair in year	< 18	31	6.2
	≥ 18	468	93.8
Age at first pregnancy in years	< 18	335	67.1
	≥ 18	164	32.9
Education	Have no formal education	301	61.7
	Have some formal education	106	32.3
	Data not recorded	30	6.0
Marital status	Married	403	80
	Divorced/separated	49	9.8
	Single	12	2.4
	Widowed	9	1.8
	Data not recorded	26	5.2

### Nutritional status of patients who underwent VVF repair

Regarding the anthropometric data of the patients, the majority of 361 (72.3%) had a weight of less than 50 kg and approximately 200 (40.1%) patients had a height of less than 150 centimeters. Among patients, the majority 362 (72.5%) had weight between 18.5 and 24.9 which can be categorized as normal weight (Table 2).

**Table 2:** Nutritional status of women who underwent successful VVF closure in Yirgalem Hamlin fistula center, Southwest Ethiopia, 2021 (*n* = 499)

Variables	Category	Frequency	Percentages (%)
Weight in kg	< 50	361	72.3
	≥ 50	138	27.7
Height in cm	< 150	200	40.1
	≥ 150	299	59.9
Body mass index (BMI) (Kg/m2)	< 18.5	115	23.0
	18.5–24.9	362	72.5
	>=25	22	4.4

### Obstetric characteristics of patients

In this study, the majority 194 (38.9%) women had parity 2–4, and more than half 215 (53.7%) had no ANC follow-up at index pregnancy. In addition, only 91 (18.2%) women were delivered within 24 h, while many women had a labor duration of 24 h. Approximately one-fourth 131 (26.3%) of the deliveries were delivered at home, and the majority 269 (53.9%) were delivered through SVD. Moreover, 393 (78.8%) of the women had had stillbirth at causative delivery (Table 3).

**Table 3:** Obstetric profiles of patients who underwent successful closure of obstetric vesicovaginal fistula repair at the Yirgalem Hamlin fistula center in 2021 (*n* = 499)

Variables	Category	Frequency	Percentage (%)
Number of parity (delivery)	1	174	34.9
	2–4	194	38.9
	≥ 5	131	26.3
Presence of ANC	Yes	268	53.7
	No	215	43.1
	Data not recorded	16	3.2
Labor duration in hours	< 24	91	18.2
	24–48	235	47.1
	≥ 48	173	34.7
Place of delivery	Home	131	26.3
	Health center	114	22.8
	Hospital	234	50.9
Mode of delivery	SVD	269	53.9
	Instrumental vaginal delivery	22	4.4.
	CS	208	41.7
Fetal outcome	Stillbirth	393	78.8
	Live	106	21.2

### Fistula characteristics of patients

Regarding the patients’ fistula characteristics, about 96 (19.2%) patients underwent VVF repair after five years; 220 (44.1%) had Goh type 2 fistula; approximately 89 (17.8%) had a large fistula (> 3 cm); and the majority 484 (97.0%) had one of the fistulae. Moreover, in this study’s findings, the majority 492 (98.6%) had no previous history of fistula repair, only 290 (58.1%) patients had intact urethral status, 412 (82.6%) had intact bladder status, and almost one-fourth of patient, 104 (20.8%) had moderate to severe vaginal scarring (Table 4).

**Table 4:** Fistula characteristics of patients who underwent successful obstetric vesicovaginal fistula repair in Yirgalem Hamlin fistula center in 2021 (*n* = 499)

Variables	Category	Frequency	Percentage (%)
Duration of fistula to repair in years	≤ 1	317	63.5
	1–5	86	17.2
	≥ 5	96	19.2
Goh Type fistula	Type 1	163	32.7
	Type 2	220	44.1
	Type 3	86	17.2
	Type 4	30	6.0
Fistula size in cm	≤ 3 cm	410	82.2
	> 3 cm	89	17.8
Number of fistulas	One	484	97.0
	Two	15	3.0
Previous history of repair	Yes	7	1.4
	No	492	98.6
Status of urethra	Intact	290	58.1
	Partially damage	179	35.9
	Completely destroyed	30	6.0
Status of bladder neck	Intact	412	82.6
	Partially or totally damaged	87	16.4
Level of vaginal scarring	None	34	6.8
	Mild	361	72.3
	Moderate to severe	104	20.8

### Patient perioperative characteristics

Among the 499 patients included in this study, the majority 475 (95.2%) had undergone a primary surgical attempt, more than one-half, 288 (57.7%) had a one layer of closure, and only 17(4.3%) had a postoperative infection (Table 5).

**Table 5:** Perioperative characteristics of patients who underwent successful obstetric vesicovaginal fistula repair at the Yirgalem Hamlin fistula center in 2021 (*n* = 499)

Variables	Category	Frequency	Percentage (%)
Surgical attempt	Primary	475	95.2
	Second	24	4.8
Layers of closure	One	288	57.7
	Two	211	42.3
Presence of infection	Yes	482	96.6
	No	17	3.4

### Magnitude of urinary incontinence after successful closure of vesicovaginal fistula repair

**Fig. 1:**
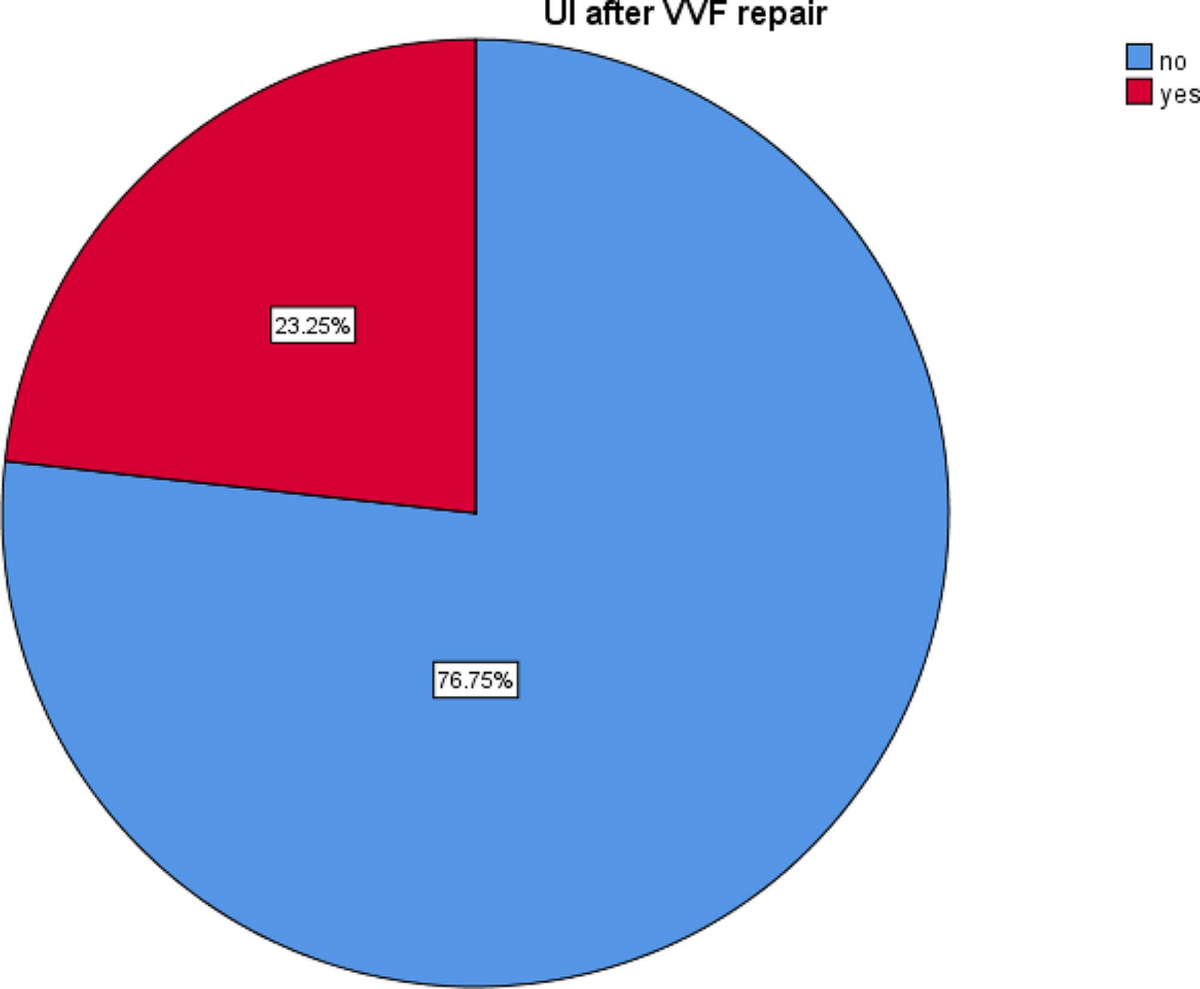
Percentages of urine incontinence among patients after successful closure of vesicovaginal fistula repair at the Yirgalem Hamlin Fistula Center in 2021 (*n* = 499)

Among the 499 study subjects in this study, 116 (23.25%) had urine incontinence after successful obstetric VVF repair (Fig. 1).

## Factors associated with urinary incontinence after successful closure of vesicovaginal fistula repair

According to the bivariate binary logistic regression analyses, 10 variables were found to be associated with the outcome variable at a p-value < 0.20. The variables collected were weight, parity, labor duration, fetal outcome, Goh type fistula, fistula size, urethral status, bladder status, vaginal scarring, and layers of closure. Among these variables, four were found to have a positive association with the outcome variable at a p-value < 0.05 according to the multivariable logistic regression. These variables were women who had Goh type 4 fistulas (AOR = 4.289; 95% CI 1.431, 12.852), a large fistula (fistula size > 3 cm) (AOR = 8.855; 95% CI 4.786, 16.382), a partially damaged urethra (AOR = 2.810; 95% CI 1.441, 5.479), a completely destroyed urethra (AOR = 5.829; 95% CI 2.094, 16.228) at a p-value of 0.009, 0.000, 0.002, and 0.001, respectively (Table 6).

**Table 6:** Bivariate and multivariate binary logistic regression analyses of factors associated with urinary incontinence among patients following successful closure of obstetric vesicovaginal fistula repair at the Yirgalem Hamlin Fistula Center in 2021 (*n* = 499)

Variables	Urine incontinence		COR (95% CI)	P value	AOR (95% CI)	P value
	No, n (%)	Yes, n (%)				
Age at repair in years						
Weight						
≥ 50 kg	112(81.2)	26(18.8)	1		1	
< 50 kg	271(75.1)	90(24.9)	0.699(0.429, 1.140)	0.151	1.661(0.899, 3.068)	0.105
Number of parties (deliveries)						
Para 1	122(70.1)	52(29.9)	1		1	
Para 2–4	151(77.8)	43(22.2)	1.497(0.936,2.393)	0.092	0.781(0.425,1.438)	0.428
Para > 5	110(84.0)	21(16.0)	2.233(1.264,3.942)	0.06	0.622(0.308,1.257)	0.186
Labor duration						
< 24	74(81.3)	17(18.7)	1			
24–48	199(84.7)	36(15.3)	0.788(0.417, 1.487)	0.461	0.728(0.334,1.587)	0.424
≥ 48	110(63.6)	63(36.4)	2.493(1.353, 4.595)	0.003	1.943(0.896,4.215)	0.093
Feta outcome						
Live birth	88(75.1)	18(24.9)	1		1	
Stillbirth	295(83)	98(17.0)	1.624(0.931,2.832)	0.087	0.965(0.477,1.953)	0.921
Goh fistula type						
Type 1	132(81.0)	31(19.0)	1		1	
Type 2	185(84.1)	35(15.9)	0.806(0.473,1.372)	0.426	0.773(0.391,1.526)	0.458
Type 3	50(58.1)	36(41.9)	3.066(1.716,5.476)	0.000	2.333(1.066,5.109)	0.045
Type 4	16(53.3)	14(46.7)	3.726(1.646,8.434)	0.002	4.289(1.431,12.852)	0.009*
Fistula size						
≤ 3 cm	351(85.6)	59(14.4)	1		1	
> 3 cm	32(36.0)	57(64.0)	10.597(6.342,17.706)	0.000	8.855(4.786,16.382)	0.000***
Urethra status						
Intact	247(85.2)	43(14.8)	1		1	
Partially damaged	123(68.7)	56(31.3)	2.615(1.663,4.112)	0.000	2.810(1.441,5.479)	0.002**
Completely destructed	13(43.3)	17(56.7)	7.512(3.404,6.318)	0.000	5.829(2.094,16.228)	0.001**
Bladder neck status						
Intact	322(80.6)	80(19.4)	1		1	
Partially or totally damaged	51(58.6)	36(41.4)	2.929 (1.792, 4.789)	0.000	1.579(0.780,3.198)	0.205
Vaginal scarring						
None	30(88.2)	4(11.8)	1		1	
Mild	287(79.5)	74(20.5)	0.2312(0.076, 0.708)	0.010	1.887(0.518,6.878)	0.336
Moderate to severe	66(63.5)	38(35.5)	0.448(0.279,0.719)	0.001	2.166(0.549,8.549)	0.270
Layers of closure						
Two	174(82.5)	37(17.5)	1		1	
One	209(72.6)	79(27.4)	1.778(1.146,2.758)	0.010	0.288(0.154,0.533)	0.047

kg = kilograms; cm = centimeter; COR = crude odds ratio; AOR = adjusted odds ratio; 1 = reference category; p values at: *p < 0.05, **p < 0.01, ***p < 0.001, N = number, while (%) represents percentages

## Discussion

The study findings showed that 23.2% (*p* = 23.2%; 95% CI 20.3, 27.9) of patients had UI following successful closure of obstetric VVF repair, and women who had a Goh type 4 fistula, a fistula size > 3 cm, a partially damaged urethra, or a completely destructed urethral status were found to have a positive association with UI following successful closure of obstetric VVF repair at the Yirgalem Hamlin Fistula Center.

In this study, the magnitude of UI following successful closure of obstetric VVF repair was in line with that reported in Pakistan (24.5%) [12], in Malawi (24%) [24], and Ethiopia (Bahir Dar Hamlin Fistula Center) (24.8%) [27]. The plausible explanations can be patients’ characteristics, a comparable surgeon’s experience, for example, in Bahir Dar Hamlin Fistula Center, and a comparable levels of sociodemographic status.

However, this percentage was greater than that reported in previous studies conducted in India (18.5%) [21], the DRC (4.3%) [13], Guinea (13.36%) [11], Burkina Faso (11.7%) [25], and Ethiopia (Jimma Specialized Hospital) (8.9%) [8]. This difference might be due to the causes of VVF, the setting difference for instance, in DRC, where data were collected from 3 fistula centers, unlike this study [13], and a longer duration of the period (10 years) used to collect data in Guinea [11], and the difference in patient individual characteristics.

In contrast, this study’s findings were lower than those of previous studies conducted in Bangladesh (37.7%) [22], Indonesia (40%) [23], and Niger (63.9%) [26]. This difference might be due to differences in the study period; for instance, in Bangladesh, the data were collected between 2008 and 2010 [22], whereas in Niger, the data were collected between 2011 and 2014 [26], and the smallest sample (10 VVF patients) was used in Indonesia [23].

In addition, Goh type 4 fistulas were found to be significantly positively associated with UI following successful closure of obstetric VVF repair. In this study, women who had Goh type 4 fistulas were approximately 4 times more likely to have UI after successful closure of obstetric VVF repair compared to women who had Goh type 1 fistulas. This finding was supported by a study done in Guinea [11], and Ethiopia (Bahir Dar Hamlin fistula center) [27]. The Goh type of fistula is pivotal in being the risk factor for UI after anatomical closure of obstetric VVF repair [33]. This might be due to the fact that the location of the fistula hole near the urethral meatus or hymen largely affects the urinary system functions, which contributes to the risk of UI following successful closure of obstetric VVF repair [34].

In this study, fistula size was also shown to be associated with UI following successful closure of obstetric VVF repair. This study demonstrated that women who had large fistulas (fistula size > 3 cm) were approximately 9.0 times more likely to have UI after successful closure of obstetric VVF repair than women who had a fistula size ≤ 3 cm. This finding was similar to previous findings in India [21] and Pakistan [12] and to study findings from five countries (Bangladesh, Guinea, Niger, Nigeria, and Uganda) [35]. A plausible reason for this difference is that large fistulas are difficult to create via tension-free surgical repair and can affect the external urinary meatus and bladder capacity.

Moreover, this study revealed that urethral status was a significant factor associated with UI following the successful closure of VVF repair. Women who had a partially damaged urethra or a completely destructed urethra were 2.8 and 5.8 times more likely to have UI following successful closure of obstetric VVF repair, respectively, than women who had an intact urethra. This finding was supported by a study performed in Guinea [11, 29]. This study finding is also supported by systematic reviews conducted in developing countries [36] and by studies conducted in five countries (Bangladesh, Guinea, Niger, Nigeria, and Uganda) [35]. Kayondo, M., et al. in Uganda also reported that urethral involvement was a significant factor for UI following succesful VVF repair [37]. This is because the urethra is a pivotal structure for controlling the urinary system. If the urethra structure of VVE is largely damaged, the women will be vulnerable to UI following the successful closure of obstetric VVF repair.

## Strengths and limitations of the study

This study has the following strengths: One of the strengths of this study was the review of patients over five years, which provided an accurate magnitude of UI during the study period. This study was also the first recent study on the determinants of UI after successful obstetric VVF repair in Ethiopia.

This study also has the following limitations: A major limitation of this study was that UI after successful obstetric VVF repair was determined retrospectively by reviewing the patient’s medical charts and operation logbooks without directly interviewing the patients. This leads to difficulty in obtaining all the needed information due to the absence of record data from medical charts and operation logbooks. The second limitation was that since the study was conducted at a single fistula center, it was difficult to generalize the findings to other fistula centers in Ethiopia.

## Conclusions

The magnitude of UI after successful closure of obstetric VVF repair was above the WHO recommendation. This study demonstrated that patient fistula characteristics, such as Goh type 4 fistulas, large fistula size, partially damaged urethra, and completely damaged urethra were found to be significant contributing factors to UI following successful closure of obstetric VVF repair. The findings of the present study will serve as a guide for policy makers or experts in the management principle of post-fistula repair UI to prevent or reduce UI following the successful closure of VVF repair. Hence, interventions are necessary to prevent or reduce post-fistula repair UI following the successful closure of VVF repair.

ANC: antenatal care; AOR: adjusted odds ratio; CI: confidence interval; COR: crude odds ratio; CS: cesarean section; DRC: Democratic Republic of Congo; SD: standard deviation; SSA: sub-Saharan Africa; SVD: spontaneous vaginal delivery; UI: urinary incontinence; VVF: vesicovaginal fistula; VVFs: vesicovaginal fistulas; WHO: World Health Organization.

## Data Availability

The datasets used and/or analyzed during the current study are available from the author upon reasonable request.
